# Revision of the key characters for the *Thricops nigrifrons* species-group (Diptera, Muscidae)

**DOI:** 10.3897/zookeys.71.788

**Published:** 2010-12-14

**Authors:** Nikita Vikhrev

**Affiliations:** Zoological Museum of Moscow University, Bolshaya Nikitskaya 6, Moscow, 125009, Russia (ZMMU)

**Keywords:** *Thricops nigrifrons*, *Thricops longipes*, Muscidae, key

## Abstract

An analysis of key characters for the separation of Thricops nigrifrons and Thricops longipes (Diptera, Muscidae) is given. A revised key for Thricops nigrifrons and related species, including two species recently described from the Caucasus, is proposed.

## Introduction

The Thricops nigrifrons species-group is characterized by: long-plumose arista; dark body; holoptic head in male; absence of apical spur on *t3* and of apical spurs on *t1* in males; *t2* without *pv* or *v* seta(e); lower margin of face not projecting; 4 postsutural *dc*; unmodified fore tarsomeres in male; mid tarsomeres 3–4 each with a row of pale *pv* setulae in male (except Thricops dawkinsi); the male terminalia are similar (Thricops semicinereus - type).

Thricops nigrifrons (Robineau-Desvoidy, 1830) and Thricops longipes (Zetterstedt, 1845) are widespread in the Palearctic region. Nevertheless Hennig (1962), d’Assis-Fonseca (1968), Gregor et al. (2002) and Savage (2003) have given different and often contradictory recommendations on how to separate these species. Recently two new related species have been described from the Russian Caucasus, Thricops tomkovichi Vikhrev, 2009 and Thricops dawkinsi Vikhrev, 2009 (Vikhrev and Sorokina 2009), and this has necessitated a revision of the characters for the separationof Thricops nigrifrons and Thricops longipes.

The examined material is restricted to Eastern Europe, Transcaucasian region and Siberia, and do not include specimens collected in Western Europe. However, the proposed key characters are expected to be suitable for west-european specimens as well, because there is no gap in natural habitats of Thricops nigrifrons and Thricops longipes in Europe. Another reason is the fact that d’Assis-Fonseca (1968) came to the same main conclusions based on the investigation of the material from British Islands.

## Material and methods

This analysis is based on the following material:

Thricops dawkinsi Vikhrev, 2009 – 23 ♂♂ and ♀♀. **Russia**:Karachay-Cherkessia, Krasnodar, North Ossetia. Holotype in Zoological Museum of Moscow University, Moscow, (ZMMU), paratypes in ZMMU, Zoological Institute, St. Petersburg (ZIN), and the Natural History Museum, London (BMNH).

Thricops longipes (Zetterstedt, 1845) – 140 ♂♂ and ♀♀. **Estonia. Russia (European)**: Arkhangelsk, Chelyabinsk, Karachay-Cherkessia, Karelia, Komi, Krasnodar, Moscow, Murmansk, Novgorod, St-Petersburg, Ulyanovsk. **Russia (Asian)**: Altai Rep (=Gorno-Altai), Khanty-Mansi, Krasnoyarsk, Novosibirsk, Tomsk, Yamalo-Nenets (ZMMU and ZIN).

Thricops nigrifrons (Robineau-Desvoidy 1830) – 150 ♂♂ and ♀♀. **Estonia. Turkey:** Bolu prov. **Russia (European)**: Chelyabinsk, Moscow, St Petersburg, Vladimir, Yaroslavl. **Russia (Asian):** Krasnoyarsk, Novosibirsk, Tomsk (ZMMU and ZIN).

Thricops tomkovichi Vikhrev, 2009 – 62 ♂♂ and ♀♀. **Russia**:Karachay-Cherkessia, Krasnodar. Holotype in (ZMMU), paratypes in ZMMU, ZIN and BMNH.

Morphological structures are abbreviated as: *f1*, *t1*, *f2*, *t2*, *f3*, *t3* = fore, mid, hind, femur or tibia; *ac* = acrostichal setae; *dc* = dorsocentral setae; *a*, *p*, *d*, *v* = anterior, posterior, dorsal, ventral seta(e).

## Notes on the identification of Thricops nigrifrons and Thricops longipes

On several occasions colleagues have expressed doubts that Thricops nigrifrons could be reliably separated from Thricops longipes. I have shared these doubts too, but currently I am convinced that a reliable (and rather easy) separation is possible. Let us first consider the identification characters proposed by Hennig (1962), d’Assis-Fonseca (1968), Gregor et al. (2002) and Savage (2003).

**Table:** 

1	The longest aristal hairs distinctly longer than width of postpedicel	Thricops nigrifrons
–	The longest aristal hairs slightly longer than width of postpedicel	Thricops longipesThis character was used as the main one by all the cited authors except for Gregor et al. (2002), although the estimation of length and wording differ. I agree that the aristal hairs are somewhat longer in Thricops nigrifrons, but I disagree with using this as the main character: the difference is very fine and the character is variable, with overlapping taking place. Gregor et al. (2002) gave the following measurements: Thricops nigrifrons – longest aristal hairs 1.05–1.30 times as long as width of postpedicel; Thricops longipes – 0,85–1.15 times. Instead of the length of aristal hairs Gregor et al. (2002) proposed the following wording:
–	Female: long aristal hairs reaching apical third of arista	Thricops nigrifrons
–	Female: long aristal hairs not reaching apical third of arista	Thricops longipesI have not found this alternative to be more reliable or easier in use than the previous one.
2	Male: upper frons with several proclinate setulae	Thricops nigrifrons
–	Male: upper frons with all setulae reclinate	Thricops longipesFirst proposed by Hennig (1962), used by Gregor et al. (2002) as the only character for males, used as the main character by Savage (2003). I agree with this character, but these upper frontal setulae are fine and often partly or even completely broken, especially in specimens mounted from alcohol.
3	Male with *p* and *v* setulose hairs on basal half of *f3* hardly longer than depth of femur	Thricops nigrifrons
–	Male with *p* and *v* setulose hairs on basal half of *f3* quite twice as long as depth of femur	Thricops longipesUsed by d’Assis-Fonseca (1968), but the other authors excluded this character from their keys. According to the descriptions given by Savage (2003): in Thricops longipes “*f3* … *p* and *v* surfaces covered with long hairs, longer than depth of femur”; in Thricops nigrifrons “*f3* … *p* and *v* surfaces covered with setae of variable length, as long to much longer than depth of femur”. I suppose that the source of the misunderstanding is that the fine hairs on the *p* and *v* surface of *f3* are not homogeneous. In Thricops longipes, these hairs really are evenly long, at least twice as long as femoral width, but in Thricops nigrifrons the hairs on the *p* surface are rather long, usually about 1–1.5 times as long as femoral width, but on the *v* surface they are short, especially in basal half of femur where the hairs are 0.5–1 times as long as femoral width. Thus, the fine hairs on *f3* are distinctly longer in Thricops longipes than in Thricops nigrifrons, but the difference is the most obvious in a comparison of the *v* hairs in the basal half of *f3*, for which the hind femur needs only to be observed in lateral view. Among about 150 male specimens examined by me, this character was always reliable and correlated with other characters. No specimens with an intermediate development of *f3* setulae were found.
4	Male *t3* with *pv* present in apical 1/2	Thricops nigrifrons
–	Male *t3* with *pv* present in apical 2/3	Thricops longipesProposed by Hennig (1962), but excluded by later authors. I agree with the exclusion of this variable character.
5	Male notopleuron bare	Thricops nigrifrons
–	Male notopleuron with a few setulae	Thricops longipesProposed by Savage (2003). In fact the notopleuron is setulose on the anterior part in both species. The notopleuron on the surface between the anterior and posterior setae is almost always bare in Thricops nigrifrons and usually setulose in Thricops longipes, but bare in a quarter of the examined specimens. This may be used as an additional character only.
6	Male: posterior part of scutum in posterior view densely dusted, without median vitta, with a pair of subshining narrow submedian vittae laterad to *dc* rows	Thricops nigrifrons
–	Male: posterior part of scutum in posterior view mostly shining black, with a wide black median vitta	Thricops longipesProposed by Vikhrev and Sorokina (2009), this character separates all examined specimens.
7	Male abdomen with the median vitta on tergite 3 inconspicuous	Thricops nigrifrons
–	Male abdomen with a conspicuous black median vitta on tergite 3	Thricops longipesProposed by Vikhrev and Sorokina (2009). The trace of a narrow and less dusted median vitta may be present in Thricops nigrifrons, but otherwise this character separates all examined specimens.
8	Male body length usually 7–7.5 mm, rarely 6–8 mm	Thricops nigrifrons
–	Male body length usually 8.5–9 mm, rarely 6.5–9.5 mm	Thricops longipes
–	Female body length usually 6.5–7.5 mm	Thricops nigrifrons
–	Female body length usually 7.5–9 mm	Thricops longipesIn spite of rare cases of overlapping, this character is at least as reliable as, and much easier to use than the width of the aristal hairs. The body size difference was also mentioned by Hennig (1962) and Savage (2003). It should be noted that this character works for the forest zone where both species are present, but not for the extreme northern populations of Thricops longipes from the tundra zone, where *nigrifons* has not been recorded. Specimens collected near Vorkuta (67.5°N) have a body size 6–8 mm only.
9	Female: postsutural part of scutum in posterior view with the median vitta indistinct, or if more or less distinct then narrow, widened only posteriorly	Thricops nigrifrons
–	Female: postsutural part of scutum in posterior view with the undusted median vitta distinct, uniformly wide throughout, occupying all the area between acrostichal rows	Thricops longipesProposed by d’Assis-Fonseca (1968), but with a misprint, so the indistinct vitta was wrongly ascribed to Thricops longipes and the distinct one to Thricops nigrifrons. Probably because of this, no one else has drawn attention to this reliable character, which separates all the females I have examined (Fig. 1).
10	Female abdomen with the median stripe narrow, often absent	Thricops nigrifrons
–	Female abdomen with a broad median stripe	Thricops longipesAgain proposed by d’Assis-Fonseca (1968), and again with the characters for Thricops longipes and Thricops nigrifrons transposed. Usually present in Thricops longipes, present or absent in Thricops nigrifrons. I think it is better to exclude this character.
11	Female: *t3* with only 2 *ad* setae	Thricops nigrifrons
–	Female: *t3* with 3–4 *ad* setae	Thricops longipesUsed by Hennig (1962) and Savage (2003). Correct in the vast majority of specimens.
12	Female: dusting on thorax and abdomen yellow with a slight brown tint	Thricops nigrifrons
–	Female: dusting on thorax and abdomen grey with a slight yellow tint	Thricops longipesProposed by Savage (2003). I agree that usually Thricops nigrifrons has more yellowish dusting while Thricops longipes is more greyish, but the reverse situation may also occur. Personally I find this character difficult to use and prefer not to include it in the key.

**Figure 1.**
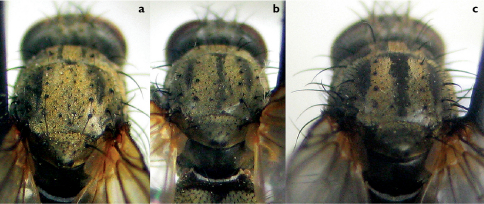
Female scutum in posterior view. **a** Thricops nigrifrons without median vitta **b** Thricops nigrifrons with narrow median vitta **c** Thricops longipes.

### Distribution

The distribution of these species in mountain areas seems sporadic and there aren’t enough reliable records. In the Austrian Alps, in the Oetz Valley, both species overlap at about 1500 m asl. Below this level, in the coniferous and broad-leaf forest zone, Thricops nigrifrons is found; above it, in the upper forest zone and above the tree-line, only Thricops longipes is found (A.C. Pont, pers. comm.). In the Russian Caucasus (Krasnodarsky Kray and Karachay-Cherkessia) Thricops longipes is found at the altitudes 1800–2500 m asl., while Thricops nigrifrons is not found. In the mountain area in Turkey, Bolu prov., 40.6N 31.8E Thricops nigrifrons is found at the altitudes 1450–1950 m asl., while Thricops longipes is not found. In European Russia Thricops nigrifrons is common in the area between Moscow and St. Petersburg (55–60°N), but absent or at least rare in Karelia at 65°N. The southern border of distribution of Thricops longipes seems to be the northern part of Moscow region (56°N), it is common in the northern coniferous forest zone (taiga) and is still the dominant species in the tundra around Vorkuta (67.5°N). In Abisko National Park (North Sweden, 68°N), with birch forest and mountain tundra (A.C. Pont, pers. comm.) and in birch-willow forest in Murmansk (69°N) only Thricops longipes was found.

### Key for the Thricops nigrifrons species-group

#### Males

**Table:** 

1	*f2* with a comb of 3–4 long and strong setae on *p-pv* surface at base and the fine setae in *av* and *pv* rows 1.5–2 times as long as *f2* width. Legs at least partly yellowish (tibiae) or both tibiae and femora yellow. *t3* with a comb of ventral consisting of 3–4 long curved setae. Caucasus	2
–	*f2* without such a comb of setae on *p-pv* surface at base and the setae in *pv* and *av* rows short, at most as long as femoral width. Legs entirely black. *t3* with 1–2 shorter ventral preapical setae. Palearctic, including Transcaucasian region	3
2	Femora black, tibiae more or less darkened basally. Mid tarsomeres 3–4 each with a row of pale *pv* setulae. Fore tarsus on *p*-surface with fine hairs that are 1.5 times as long as tarsal width	Thricops tomkovichi Vikhrev
–	All femora and tibiae yellow, at most fore femur slightly darkened. Mid tarsomeres 3–4 each with the row of pale *pv* setulae reduced. Fore tarsus with the *p*-hairs not longer than tarsal width	Thricops dawkinsi Vikhrev
3	In lateral view, *f3* on basal half with fine hairs on *v* surface at most as long as femoral width, much shorter than the strong *av* setae (the hairs on *p* surface at most 1.5 times as long as femoral width). In posterior view postsutural part of scutum densely yellowish-grey dusted without a median vitta between acrostichals (a pair of vittae present laterad to *dc* rows). Abdomen with the median vitta on tergite 3 inconspicuous, at most a narrow trace of a vitta present. Frons with both pro- and reclinate setulae on upper half. Secondary characters: body length usually 7–7.5 mm, rarely 6–8 mm; ground setulae absent between the two notopleural bristles, longest aristal hairs 1.05–1.30 times as long as width of postpedicel	Thricops nigrifrons (Robineau-Desvoidy)
–	In lateral view, *f3* on basal half with fine hairs on *v* surface about twice as long as femoral width, about as long as the strong *av* setae (these hairs on *p* surface at least twice as long as femoral width). In posterior view, postsutural part of scutum subshining black with only thin greyish dusting, consisting of two vittae restricted to areas between *ac* rows and slightly beyond *dc* rows, median vitta between *ac* rows always present. Abdomen with a black subshining median vitta on tergite 3 wide and distinct on at least anterior 2/3 of tergite. Frons with all setulae on upper half reclinate. Secondary characters: body length usually 8.5–9 mm, rarely 7.5–9.5 mm, ground setulae usually (in 75% specimens) present between the two notopleural bristles, longest aristal hairs 0.85–1.15 times as long as width of postpedicel	Thricops longipes (Zetterstedt)

#### Females

**Table:** 

1	Legs entirely black. Palearctic, including Transcaucasian region	2
–	Legs partly or entirely yellow. Caucasus	3
2	Postsutural part of scutum in posterior view with the median vitta indistinct (Fig. 1a), or if more or less distinct then narrow, sometimes slightly widened posteriorly (Fig. 1b). *t3* with only 2 *ad* setae. Longest aristal hairs 1.05–1.30 times as long as width of postpedicel. Body length usually 6.5–7.5 mm	Thricops nigrifrons (Robineau-Desvoidy)
–	Postsutural part of scutum in posterior view with the undusted median vitta distinct, uniformly wide throughout, occupying all the area between *ac* rows (Fig. 1c). *t3* with 3–4 *ad* setae, the additional seta(e) often short. Longest aristal hairs 0.85–1.15 times as long as width of postpedicel. Body length usually 7.5–9 mm	Thricops longipes (Zetterstedt)
3	Femora black, tibiae darkened basally	Thricops tomkovichi Vikhrev
–	Femora and tibiae yellow	Thricops dawkinsi Vikhrev

## References

[B1] d’Assis-Fonseca ECM (1968) Diptera Cyclorrhapha Calyptrata, Muscidae. Handbook for the Identification of British Insects 10, 4(b). Royal Entomological Society, London, 118 pp.

[B2] Gregor F Rozkosny R Bartak M Vanhara J (2002) The Muscidae (Diptera) of Central Europe. Folia Facultatis Scientiarum Naturalium Universitatis Masarykianae Brunensis, Biologia 107:1-280

[B3] Hennig W (1955–1964) Family Muscidae. In: Lindner E (Ed) Die Fliegen der Palaearktischen Region 63b. E. Schweizerbart, Stuttgart, 1110 pp.

[B4] Savage J (2003) Revision of the genus Thricops Rondani (Diptera: Muscidae). Insect Systematics and Evolution Supplement 61:1-143

[B5] Vikhrev N Sorokina V (2009) Faunistic records of Thricops Rondani (Diptera, Muscidae) from Russia with description of two new species. Euroasian Entomological Journal 8 (3):341-350

